# Evaluation of root and canal morphology of maxillary permanent first molars in an Emirati population; a cone-beam computed tomography study

**DOI:** 10.1186/s12903-020-01269-2

**Published:** 2020-10-07

**Authors:** Eman Al Mheiri, Jahanzeb Chaudhry, Salma Abdo, Rashid El Abed, Amar Hasan Khamis, Mohamed Jamal

**Affiliations:** 1 Department of Endodontics, Hamdan Bin Mohammed College of Dental Medicine, Mohammed Bin Rashid University of Medicine and Health Sciences, Building 14, Dubai Health Care City, Dubai, United Arab Emirates P.O.Box: 505055,; 2 Dental Department, Dubai Health Authority, Dubai, United Arab Emirates; 3 Endodontic department, Al Ain Dental Centre, Seha Ambulatory Healthcare Services, Al Ain, Abu Dhabi, United Arab Emirates; 4 Dental department, Mediclinic, Dubai, United Arab Emirates

**Keywords:** Emirati population, Second mesiobuccal canal, Canal configuration, Cone-beam computed tomography

## Abstract

**Background:**

The aim of this study was to analyze the root and canal morphology of the maxillary permanent first molars in an Emirati population using cone-beam computed tomography (CBCT).

**Methods:**

Two hundred and sixty-one CBCT scans were acquired. The data were extracted and anonymized to remove all patient identifiers. Two observers (an endodontic resident and an endodontist) evaluated all scans on diagnostic quality monitors.

**Results:**

The prevalence of a second mesiobuccal canal (MB2) was 80.1% in all examined samples. Type II Vertucci classification, was the most common canal configuration (59%) in the mesiobuccal root, followed by Types I (19.9%) and IV (15.3%), while Type III was the least common (5.7%). Types I, II, and IV were significantly more common in the 21–40-year age group (*P* < 0.001), while Type III was observed significantly more often in the < 20-year age group (*P* < 0.001). No significant effect of gender on the prevalence of Vertucci classification in the mesiobuccal root of maxillary first molars (*P* = 0.74) was found. Analysis of bilateral symmetry showed that 80% teeth had perfect bilateral symmetry, whereas 20% were asymmetrical. Type II canal configuration showed the highest prevalence of bilateral symmetry (48.7%), followed by Type I (15%) and Type IV (10%), while Type III showed the least prevalence of symmetry (3%).

**Conclusions:**

This was the first study to analyze the prevalence of MB2 canal in an Emirati population. Our results indicate high prevalence of MB2 (80.1%) and emphasize the importance of using advanced techniques to locate the MB2 canal.

## Background

One of the most frequent causes of endodontic treatment failure is difficulty in identifying and treating all or part of root canal anatomy [1–4] . A missed canal might harbor necrotic tissues or microorganisms which can lead to persistent periapical pathology. The root and canal morphology of the maxillary first molars are frequently studied because of their complex anatomy and being one of the most common teeth to have root canal treatment [1, 5]. It is generally accepted that most maxillary first molars have 3 roots and 4 canals; various studies have reported prevalence of second mesiobuccal canal (MB2) in the range of 52–93% [1, 6–11]. Despite its high prevalence, MB2 is still difficult to find due to diffuse calcification, its narrowness and unusual location of the orifice. Missed MB2 is considered one of the most common cause of endodontic treatment failure of maxillary first molars. This has been emphasized since 1969, when Franklin et al. [1], concluded that locating MB2 increases the success of endodontic therapies in maxillary first molars. In a more recent, 5-years prospective clinical study, which included 5600 root canal-treated and retreated teeth, it was concluded that failure to locate and manage existing MB2 in maxillary first molars would detrimentally affect the prognosis of root canal treatment [5]. Several other studies, showed similar findings, with missed MB2 in maxillary first molars resulting in endodontic treatment failure [12–14]. Despite the recent advances in available diagnostic tools to evaluate maxillary first molars canal anatomy and locate MB2, such as the dental operating microscope (DOM) and cone-beam computed tomography (CBCT), the basic knowledge of maxillary first molar morphology and the prevalence of MB2, its variations and the effects of ethnicity on such variations is essential.

Several methods such as tooth clearing and staining, radiological methods (intraoral radiographs and CBCT), grinding and sectioning, and clinical observations [14, 15] have been used to study root and canal morphologies. CBCT allows the three-dimensional assessment of dental and maxillofacial structures by providing an excellent non-invasive model and hence, is indispensable for the analysis of root and canal anatomies [10, 16, 17]. Several studies have successfully used CBCT to analyze root and canal anatomies of the maxillary molars in different populations and ethnic groups with reported prevalence of MB2 in the range of 40–80% [10, 18–25]. After an extensive review of the literature, we determined that no study has analyzed the anatomy of maxillary first molars in the Emirati population. Such study if available will provide valuable information on possible variations of canal anatomy in maxillary first molars and prevalence of MB2 for dentists treating this population. Such information will aid in the decision-making process before, during and after endodontic treatment. It will also encourage dentists to use advanced technologies such DOM and CBCT or to refer to specialists if necessary. Therefore, the aim of this study is to describe the root and canal morphology of maxillary permanent first molars in an Emirati population using CBCT.

## Methods

### Sample collection

The institutional review board approvals were obtained from the concerned committees of the Mohammed Bin Rashid University of Medicine and Health Sciences and Abu Dhabi Health Authority to conduct this retrospective study. CBCT scans of patients who were treated at a community dental center between 2017 and 2018 were obtained and analyzed. The CBCT scans were acquired using the Planmeca ProMax CBCT scanner (Planmeca Oy, Helsinki, Finland). The imaging protocol was as follows: Field of view (FOV) = 16 × 11 cm; tube peak potential = 120 kVp; tube current = 18.54 mA; time = 8.9 s; voxel size = 0.4 mm. The scans of patients who met the following inclusion criteria were included in the study: Emirati, age range 12–75 years, presence of bilateral permanent maxillary first molars, completely matured and erupted teeth. Permanent maxillary first molars with root canal fillings, posts, crowns, coronal or root resorption, extensive coronal or root caries, and/or periapical or periradicular radiolucency were excluded from the study. Two hundred sixty-one scans that met the inclusion and exclusion criteria were randomly selected, anonymized, and exported from the Abu Dhabi Health Authority database in Digital Imaging and Communication in Medicine (DICOM) format. The sample size of 261 scans was determined based on power analysis using Cochran’s test, where the number of MB2 in previous studies [26, 27] was considered the relevant difference.

### Radiographic evaluation

Two observers (an endodontic resident and an expert endodontist) evaluated all scans on an iMAC computer ([27-in. screen size with Retina 5 K display, 5120 × 2880 resolution with support for 1 billion colors, 500 nits brightness], Apple, USA) in a room with controlled lighting using the Horos DICOM viewer (horosproject.org) [28]. The root and canal morphology were assessed using the three-dimensional multiplanar reconstruction (3D MPR) tool, in which all images were examined in the axial, coronal, and sagittal planes. Furthermore, the observers determined the coronal section to be within 2 mm of cemento-enamel junction (CEJ), middle third section to be within 2 mm of mid root length (from apex to CEJ) and apical section to be at apex and 2 mm above. The two observers were trained and calibrated before the evaluation process. For observer training and calibration, a sample of CBCT scans, exhibiting all 8 types of root canal morphology in the maxillary first molars as per Vertucci classification (VC) [29], was used.

**Fig. 1:**
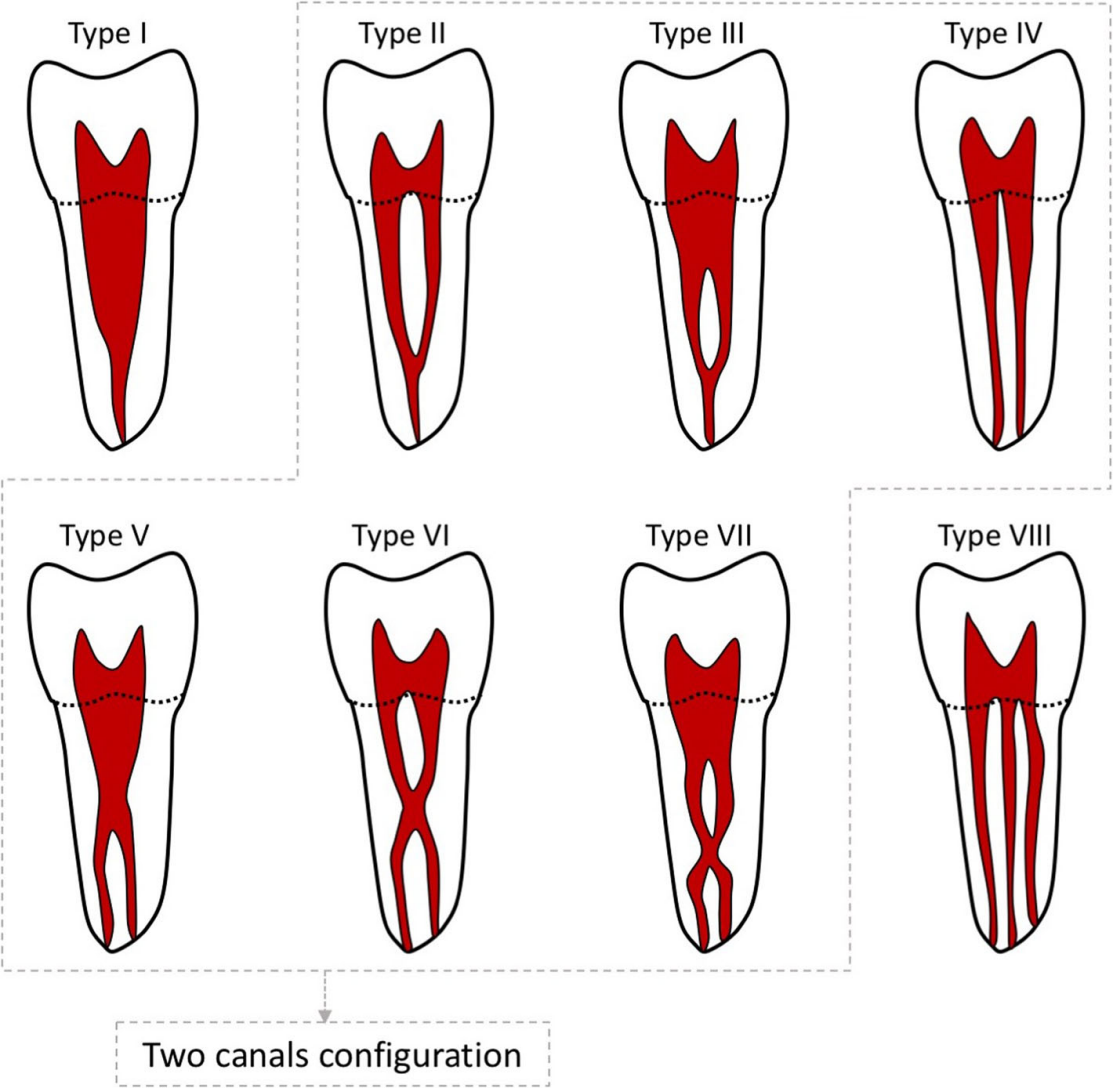
Vertucci classification of the root canal system. Type II – Type VII (enclosed in gray dashed line) represent two canal configurations

The observers recorded the number of roots and the canal morphology of each root of the maxillary first molars as per VC (Table 1, Fig. 1). MB2 is considered to be present if one of the two canal configurations of VC (Type II, III, IV, V, VI and VII) is observed in the mesiobuccal root (Fig. 1). Moreover, the symmetry in canal morphology between the right and left sides was recorded. The observers reviewed and recorded the findings twice with a gap of 30 days between the two reviews. The sequence of scans in the second review was different from that in the first review. The findings were finalized based on recordings of both the observers. Disagreements between the observers were resolved by consulting a third evaluator (expert endodontist). The data were subjected to kappa test to ensure reproducibility and reliability, and the Altman’s scale was used for interpretation. Finally, the findings were tabulated and were correlated with age and gender.

**Table 1:** Vertucci canal classification

Type	Description
Type I	A single canal extends from the pulp chamber to the apex
Type II	Two separate canals leave the pulp chamber and join short of the apex to form a single canal
Type III	One canal leaves the pulp chamber, divides into 2 within the root, and then merges to exit as a single canal
Type IV	Two separate and distinct canals extend from the pulp chamber to the apex
Type V	One canal leaves the pulp chamber and divides short of the apex into 2 separate and distinct canals with separate apical foramina
Type VI	Two different canals go the pulp chamber, merge in the body of the root, and redevise short of the apex to exit as 2 distinct canals
Type VII	One canal leaves the pulp chamber, divides and then rejoins within the body of the root, and finally re-divides into 2 distinct canals short of the apex
Type VIII	Three separate and distinct canals extend from the pulp chamber to the apex

### Statistical analysis

Data were analyzed using SPSS for Windows version 25.0 (SPSS Inc., Chicago, IL). Results were cross-tabulated to examine the dependency between variables. Statistical analysis was performed using χ2 (Chi-square) to determine of association between variables such as distribution of MB2 by gender, age or site. Kappa test was used to test inter- and intra-rater reliability. Frequency tables’ bar and lines graphs were used as descriptive statistics. A *P*-value of less than 0.05 was considered significant in all statistical analysis.

## Results

As planned, the two observers reviewed the 261 CBCT scans independently with focus on left and right maxillary first molars. As only CBCT scans of patients who have bilateral maxillary first molar were included, therefore a total of 522 M were studied. The kappa test indicated moderate concordance within the same examiner (Kappa = 0.44) and good concordance among the 2 examiners (Kappa = 0.61). The expert opinion was required in 58 out of 261 scans.

Of the 261 patients, 145 (55.6%) were females and 116 (44.4%) were males. The age of patients ranged from 12 to 71 years; 28% were younger than 21 years, 52.1% were between 21 and 40 years and 19.1% were older than 40 years (Table 2).

**Table 2:** Demographic Data

Age			Gender	
< 20	21–40	> 40	Female	Male
73 (28%)	136 (52.1%)	52 (19.9%)	145 (55.6%)	116 (44.4%)

**Fig. 2:**
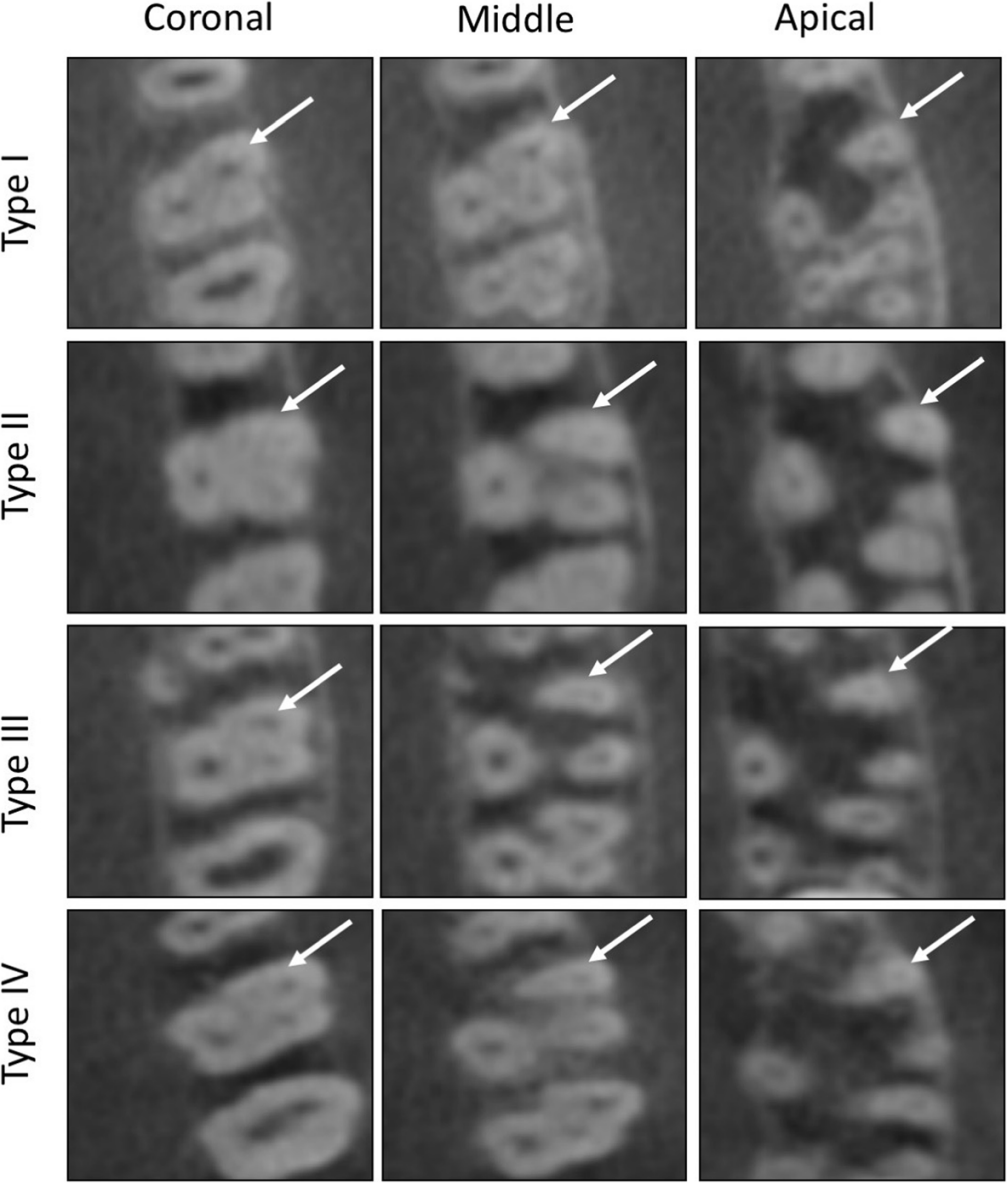
Axial view of CBCT scans at different root levels showing the different canal configurations observed in the mesiobuccal root of maxillary permanent first molar. White arrows point to the mesiobuccal root

Among the studied 522 maxillary permanent fist molars, most teeth had 3 roots (98.9%), while only 0.5 and 0.6% had 2 and 4 roots, respectively. Root canal configurations of only Types I and II of the VC were detected in the palatal and distobuccal roots, of which Type I was more prevalent, while Type II was observed in only 1.2 and 2% in the palatal and distobuccal roots, respectively (Table 3). The mesiobuccal root showed a single canal configuration (Type I) in 19.9%, while the 2-canal configurations (Types II, III, or IV) were observed in 80.1%. Most mesiobuccal roots had Type II VC (59%), followed by Type I and Type IV (19.9 and 15.3%, respectively). The least common canal configuration in the mesiobuccal root was Type III (6%) (Table 3). No other types were observed in the mesiobuccal roots. Figure 2 shows an example of observed canal configurations in the mesiobuccal root.

**Table 3:** Canal configuration per root of maxillary permanent first molar

	Mesiobuccal root	Distobuccal root	Palatal root
Single canal configuration			
Type I	104 (19.9%)	512 (98%)	516 (98.8%)
Two canals configuration			
Type II	308 (59%)	10 (2%)	6 (1.2%)
Type III	30 (5.7%)	–	–
Type IV	80 (15.3%)	–	–

Analysis of prevalence of a specific canal configuration in age groups in the mesiobuccal root showed that Types I, II, and IV were observed significantly more often in the 21–40-year age group than that among other age groups (*P* < 0.001). Type III was observed significantly more often in the < 20-year age group than that in other age groups (*P* < 0.001). Further dependency analysis showed no significant effect of gender on canal configuration in the mesiobuccal root of maxillary first molars (*P* = 0.74).

**Fig. 3:**
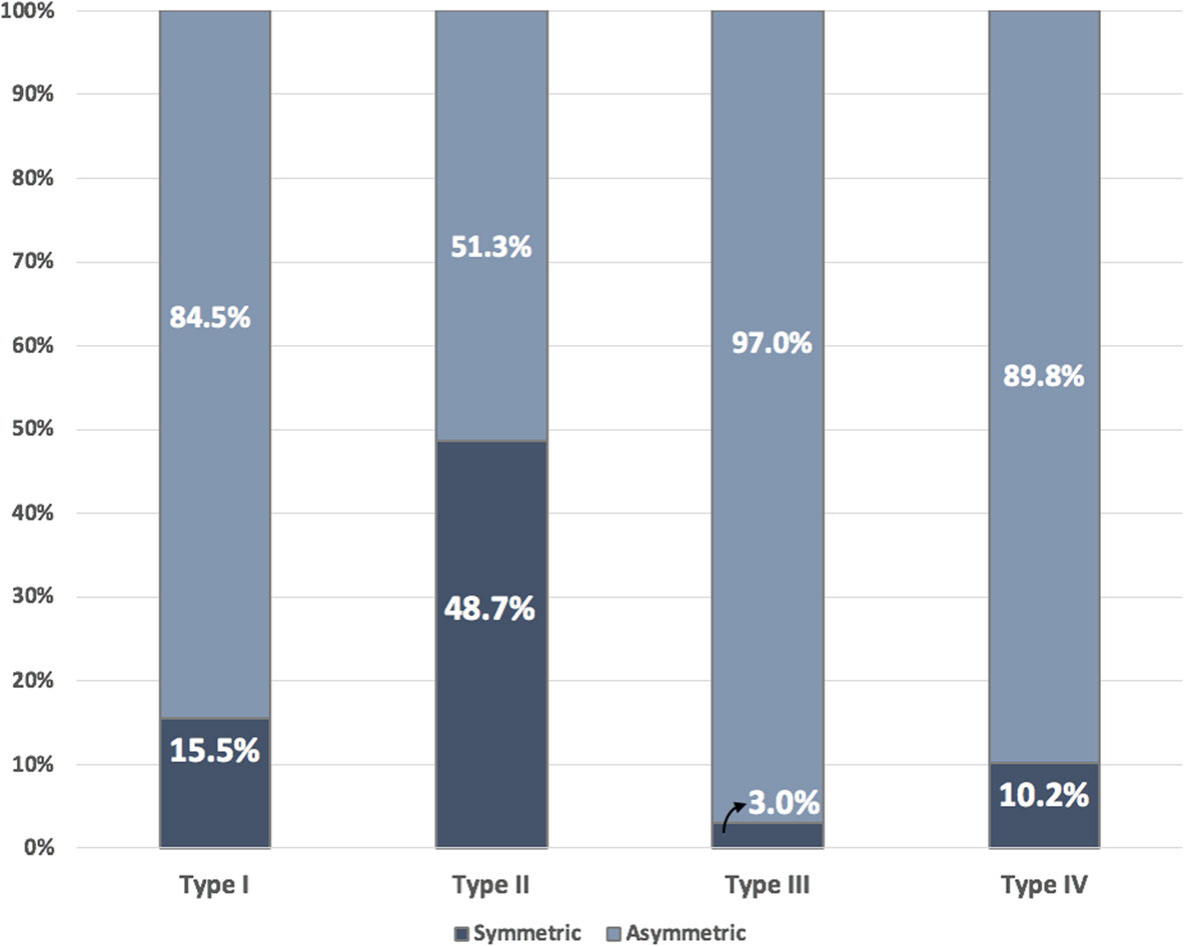
Bar chart indicating the bilateral symmetry/asymmetry of different Vertucci classification in the mesiobuccal root

The analysis of bilateral symmetry in canal configuration in the mesiobuccal root of right and left maxillary first molars showed that 80% canal configurations were bilaterally symmetrical and 20% were asymmetrical. More specifically, Type II had the highest incidence of bilateral symmetry (48.7%), followed by Type I (15%) and Type IV (10%), while Type III had the least incidence (3%) (Fig. 3).

## Discussion

Endodontic treatment of the maxillary first molars is considered a challenge due to the complex root and canal anatomies and high prevalence of MB2 canal [8, 9, 30]. Several studies have reported the importance of locating and cleaning the MB2 canal in the success of endodontic therapy [4, 5, 30]. Multiple approaches have been suggested to facilitate the localization of the MB2 canal and other anatomical variations, such as acquisition of knowledge regarding the differences in canal anatomy in different races and ethnic groups and using advanced clinical techniques such as DOM, stains, troughing with ultrasonic, and CBCT [9, 16, 31]. Ball et al. in 2013 recommended the use of CBCT in cases with unexpected complex anatomy or with difficult to locate canals [31].

In this study we examined the root and canal configuration of the maxillary first molars in an Emirati population using CBCT. Our aim is to provide information to clinicians who treat this population and to address the knowledge gap related to the root and canal morphology of the maxillary first molars in this population. Our results showed that most teeth were 3 rooted (98.9%) while only 0.4 and 0.6% had 2 and 4 roots respectively. Our results are similar to other studies which indicated that most maxillary first molars have 3 roots (range from 82 to 100%) followed by 2 roots (up to 9%) [6, 10, 11, 18–27, 32, 33]. Very few studies reported 4 rooted maxillary first molars. Neelakantan et al. reported 4 rooted maxillary first molars in 0.9% of examined teeth in Indian population [34]. Martin et al. studied the root and canal morphology of 5250 maxillary first molars using CBCT scans collected from 21 counties. They reported prevalence of 4 rooted maxillary first molar that range from 0 to 1.6% with an average of 0.2%. Most examined molars in their study had 3 roots (94%), followed by 2 roots (5.4%) and 1 root (0.4%) [35].

With regards to the canal morphology, our results showed that in the palatal and distobuccal roots, single canal configuration (Type I VC) was more prevalent, with only 1.2 and 2% of the examined teeth showed Type II VC in the palatal and distobuccal roots, respectively. These results are similar to several studies conducted in several populations, in which palatal and distobuccal root of maxillary first molars had single canal in a percentage range from 98 to 100% [6, 11, 23, 25, 27].

**Fig. 4:**
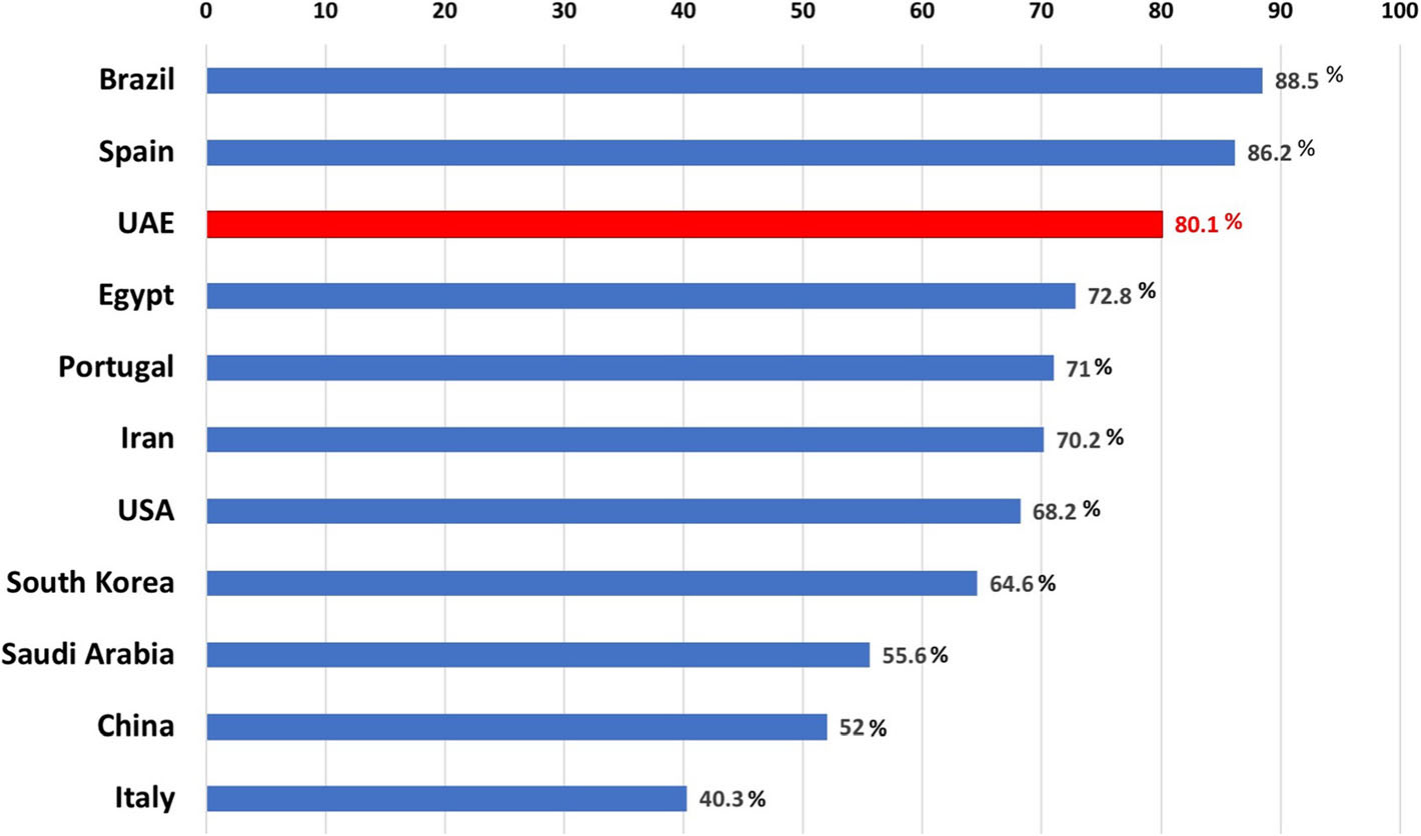
Bar chart indicating MB2 prevalence in different populations. These studies were conducted using CBCT

Our analysis for mesiobuccal root of examined maxillary first molars showed that 19.9% had single-canal configuration (Type I), while 80.1% showed 2-canal configurations (Type II, III, and IV VC). Our results are similar to those of several other studies conducted using CBCT in different populations worldwide. The prevalence of MB2 was reported to be 71% among the Portuguese population [18], 86.2% among the Spanish population [19], 88.5% among the Brazilian population [20], 55.6% among Saudi population [27], 40.3% among the Italian population [21], 72.8% among the Egyptian population [22], 68.2% among the American population [23], and 70.2% among the Iranian population [24]. Kim et al. reported the prevalence of MB2 to be 64.6% among the Korean population [25], while Zhang et al. reported a prevalence of 52% among the Chinese subpopulation [10] (Fig. 4).

In our study, most maxillary first molars (59%) exhibited the Type II VC. The prevalence of Type I and Type IV was 19.9 and 15.3%, respectively. Our results are similar to those of Perez-Heredia et al., in which they found that Type II was the most common VC (56.5%), followed by Type IV and Type I (23.2 and 13.8%, respectively) [19]. Kim et al. reported that Type IV was the most common canal configuration (40.6%), followed by Type I and II (36.4 and 20.4%, respectively) [25]. Our results showed that Type III was the least common canal configuration (6%). Moreover, Types V, VI, VII, and VIII VC were not observed. These results are in agreement with those of several similar CBCT studies, where Types VI, VII, and VIII were not observed in the mesiobuccal root of maxillary first molars [21, 22, 24, 27].

Our correlation analysis showed a significant relationship between age and the presence of MB2 canal. Types II and IV were observed significantly more often in the 21–40-year age group than in the other age groups (*P* < 0.001), while Type III was observed significantly more often in the < 20-year age group than in the other age groups (*P* < 0.001). These results indicate that as age increases, the incidence of complex canal configuration decreases. Our results are in agreement with the findings of Reis et al., which indicated an inverse correlation between age and the presence of MB2 [20]. Similar findings were reported by Zheng et al., wherein MB2 was most commonly observed in the 20–30-year age group [11]. Neaverth et al. reported the highest incidence of MB2 in the 20–40-year age group [7]. This phenomenon could be attributed to the continuous dentin deposition over time resulting in canal calcification and narrowing. Using micro-CT scanning, Oi et al. reported a decrease in the size of the pulp cavity and in canal diameter with an increase in age [36]. Our correlation analysis showed no effect of gender on the presence of MB2 canal or a certain type of canal configuration in general.

The analysis of bilateral symmetry of canal configurations in the mesiobuccal root of right and left maxillary first molars showed that 80% canal configurations were bilaterally symmetrical and 20% were asymmetrical. More specifically, Type II VC showed the highest bilateral symmetry (48.7%), followed by Type I (15%) and Type IV (10%), while Type III was the least prevalent (3%). Our results are very similar to those of Plotino et al., which showed bilateral symmetry in 79.6% teeth [21]. Guo et al. reported bilateral symmetry in 65.6% teeth [23]. Interestingly, Type I VC showed 15% symmetry implying that if a clinician finds a Type I VC (single canal configuration) on one side, there is an 85% chance that the other side would have any of the 2-canal configurations, Types II, III, or IV. Therefore, clinicians should continue their search for the MB2 canal on the other side, even if only one canal was encountered in the mesiobuccal root of the maxillary first molar on one side.

This study reported a relative high incidence of MB2 (80.1%) in an Emirati population, and it was present in the form of type II, III and IV VC. Beside the importance of this information on encouraging clinicians to search and locate MB2, clinicians should be aware of the clinical challenges associated with the different forms of MB2 and plan their root canal treatment accordingly. For example, in case MB2 was in the form of type II VC, then both canals in the mesiobuccal roots will share the same apical foramen, therefore there are high chances of iatrogenic cleaning and shaping errors, such as; over-enlarging the apical foramen, ledging, transportation and blockage of one of the canals [37]. Clinicians can avoid such errors by carefully selecting their cleaning and shaping protocol or clean and shape one of the canals to the full length while the other to the joining level [38, 39]. Another example are the clinical challenges related to MB2 in the form of type III VC, in which the MB2 orifice might not always be at the CEJ or pulp floor level but can start deeper apically. Therefore, clinicians should always scout the main canal with curved small hand file, especially if the orifice of MB2 was not found at the pulp floor level [37, 40, 41].

A possible limitation of this study is voxel size of CBCT scans, which was 0.4 × 0.4 mm. This voxel size results in lower image resolution than the image resolution obtained in comparable studies [18–20, 22, 23]. Such scans with lower resolutions could adversely affect an evaluator’s ability to read the scans accurately, especially when reading small structures, such as calcified and lateral canals [17, 42]. Bauman et al. showed that there is increase in detection rate of MB2 from 60.1 to 93.3% at voxel size of 0.4 mm to 0.125 mm [43]. Furthermore, Yan Ji et al. reported that even at 0.125 mm voxel size, it can still be challenging to detect small fine structure such as lateral canal [44].

## Conclusions

Our study is the first to analyze the prevalence of MB2 canal in Emirati subpopulation. Our results show that the prevalence of MB2 in the Emirati subpopulation is relatively high (80.1%). and emphasize the importance of searching for and using advance techniques to locate the MB2 canals in permanent maxillary first molars.

## Data Availability

The datasets used and/or analysed during the current study are available from the corresponding author on reasonable request.

## Abbreviations

3D MPR: Three-dimensional multiplanar reconstruction
CBCT: Cone-beam computed tomography
CEJ: Cemento-enamel junction
DICOM: Digital Imaging and Communication in Medicine
DOM: Dental operating microscope
MB2: Second mesiobuccal canal
VC: Vertucci classification
